# Supplementary material for: Co-Expression of CD34, CD90, OV-6 and Cell-Surface Vimentin Defines Cancer Stem Cells of Hepatoblastoma, Which Are Affected by Hsp90 Inhibitor 17-AAG
Source: Cells. 2021 Sep 29;10(10):2598. doi: 10.3390/cells10102598 (PMC8533921; doi:10.3390/cells10102598)
Supplement: Supplementary file 1 [file cells-10-02598-s001.zip › Supplementary figures.pptx]

## Slide 1
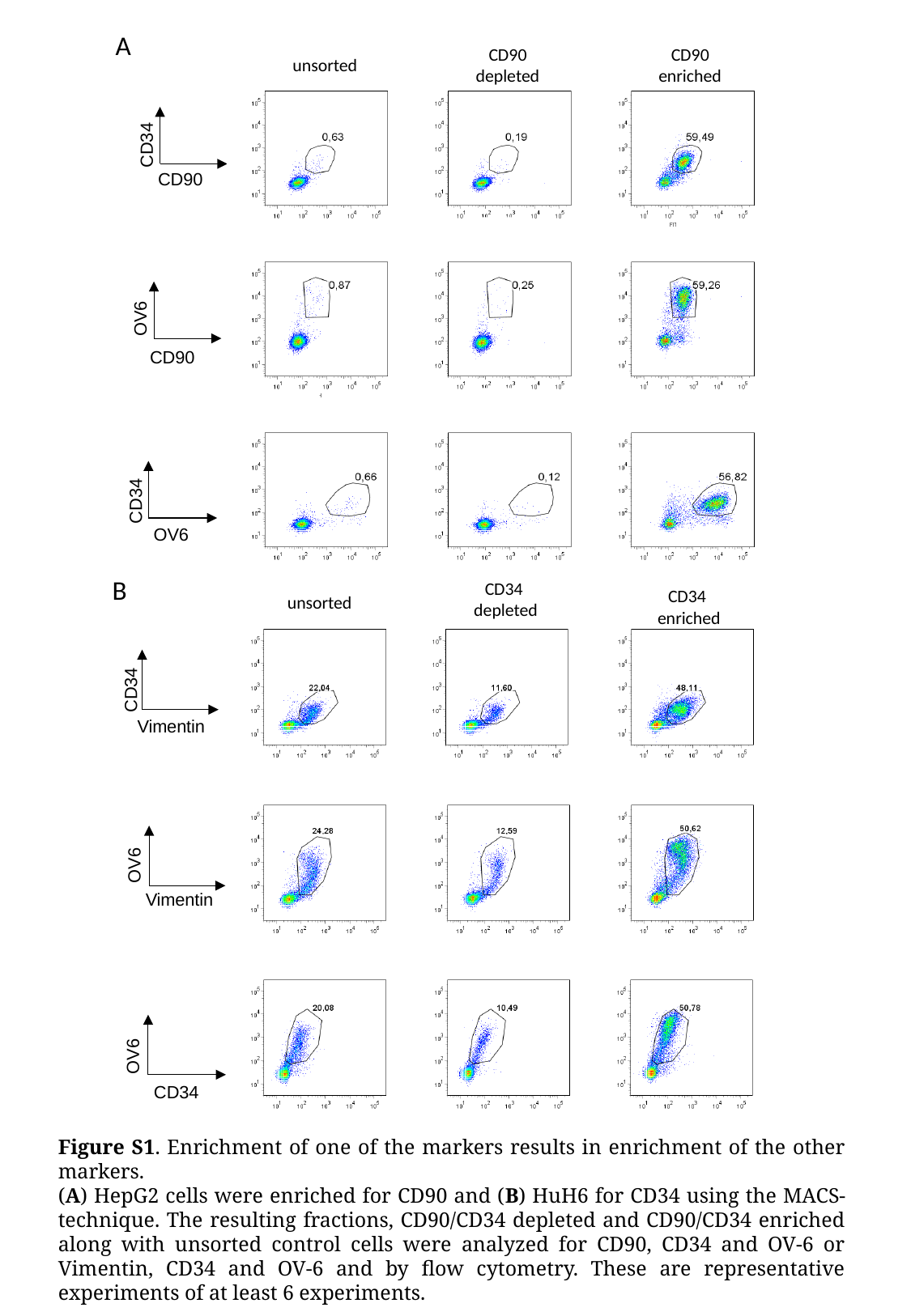

A
CD90 depleted
CD90 enriched
unsorted
CD34
CD90
OV6
OV6
CD90
CD34
B
CD34
depleted
CD34
 enriched
unsorted
CD34
Vimentin
OV6
Vimentin
CD34
OV6
Figure S1. Enrichment of one of the markers results in enrichment of the other markers.
(A) HepG2 cells were enriched for CD90 and (B) HuH6 for CD34 using the MACS-technique. The resulting fractions, CD90/CD34 depleted and CD90/CD34 enriched along with unsorted control cells were analyzed for CD90, CD34 and OV-6 or Vimentin, CD34 and OV-6 and by flow cytometry. These are representative experiments of at least 6 experiments.

## Slide 2
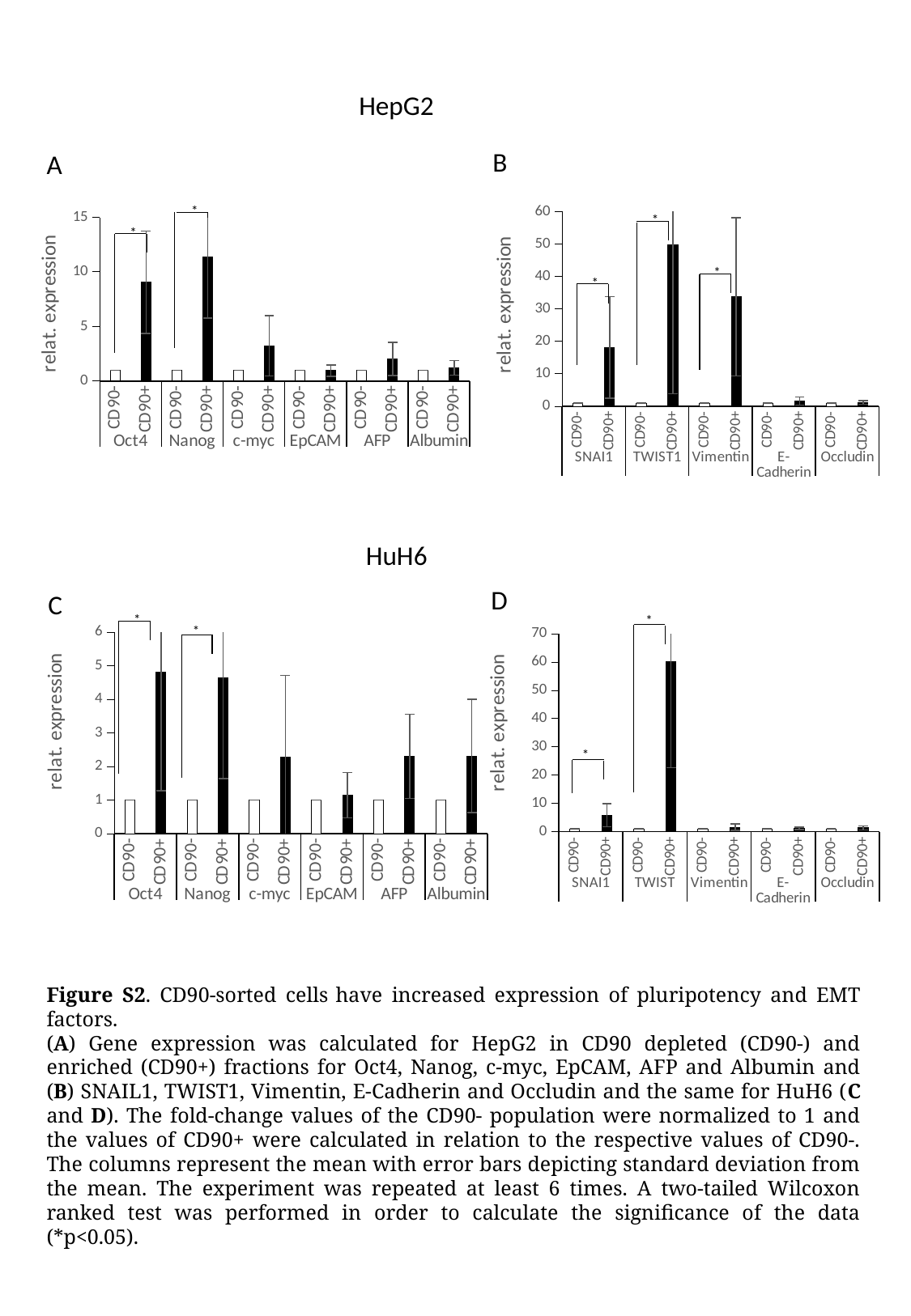

HepG2
B
A
### Chart
| Category | |
|---|---|
| CD90- | 1.0 |
| CD90+ | 9.052709924637853 |
| CD90- | 1.0 |
| CD90+ | 11.34913751846383 |
| CD90- | 1.0 |
| CD90+ | 3.222898334277019 |
| CD90- | 1.0 |
| CD90+ | 0.9542588334230936 |
| CD90- | 1.0 |
| CD90+ | 2.0308910392078476 |
| CD90- | 1.0 |
| CD90+ | 1.2044953707132688 |*
### Chart
| Category | |
|---|---|
| CD90- | 1.0 |
| CD90+ | 18.09807195354574 |
| CD90- | 1.0 |
| CD90+ | 49.64874758011179 |
| CD90- | 1.0 |
| CD90+ | 33.71923409411485 |
| CD90- | 1.0 |
| CD90+ | 1.6302753059852144 |
| CD90- | 1.0 |
| CD90+ | 1.2301810688553356 |*
*
*
*
HuH6
D
C
*
*
*
### Chart
| Category | |
|---|---|
| CD90- | 1.0 |
| CD90+ | 4.811393066595642 |
| CD90- | 1.0 |
| CD90+ | 4.631002142202102 |
| CD90- | 1.0 |
| CD90+ | 2.2921740689363284 |
| CD90- | 1.0 |
| CD90+ | 1.1464614398924122 |
| CD90- | 1.0 |
| CD90+ | 2.299826436740949 |
| CD90- | 1.0 |
| CD90+ | 2.316403460649574 |
### Chart
| Category | |
|---|---|
| CD90- | 1.0 |
| CD90+ | 5.7628687452644805 |
| CD90- | 1.0 |
| CD90+ | 60.18915840029785 |
| CD90- | 1.0 |
| CD90+ | 1.6042344230084982 |
| CD90- | 1.0 |
| CD90+ | 1.2111305847193425 |
| CD90- | 1.0 |
| CD90+ | 1.3293628651386635 |*
Figure S2. CD90-sorted cells have increased expression of pluripotency and EMT factors.
(A) Gene expression was calculated for HepG2 in CD90 depleted (CD90-) and enriched (CD90+) fractions for Oct4, Nanog, c-myc, EpCAM, AFP and Albumin and (B) SNAIL1, TWIST1, Vimentin, E-Cadherin and Occludin and the same for HuH6 (C and D). The fold-change values of the CD90- population were normalized to 1 and the values of CD90+ were calculated in relation to the respective values of CD90-. The columns represent the mean with error bars depicting standard deviation from the mean. The experiment was repeated at least 6 times. A two-tailed Wilcoxon ranked test was performed in order to calculate the significance of the data (*p<0.05).

## Slide 3
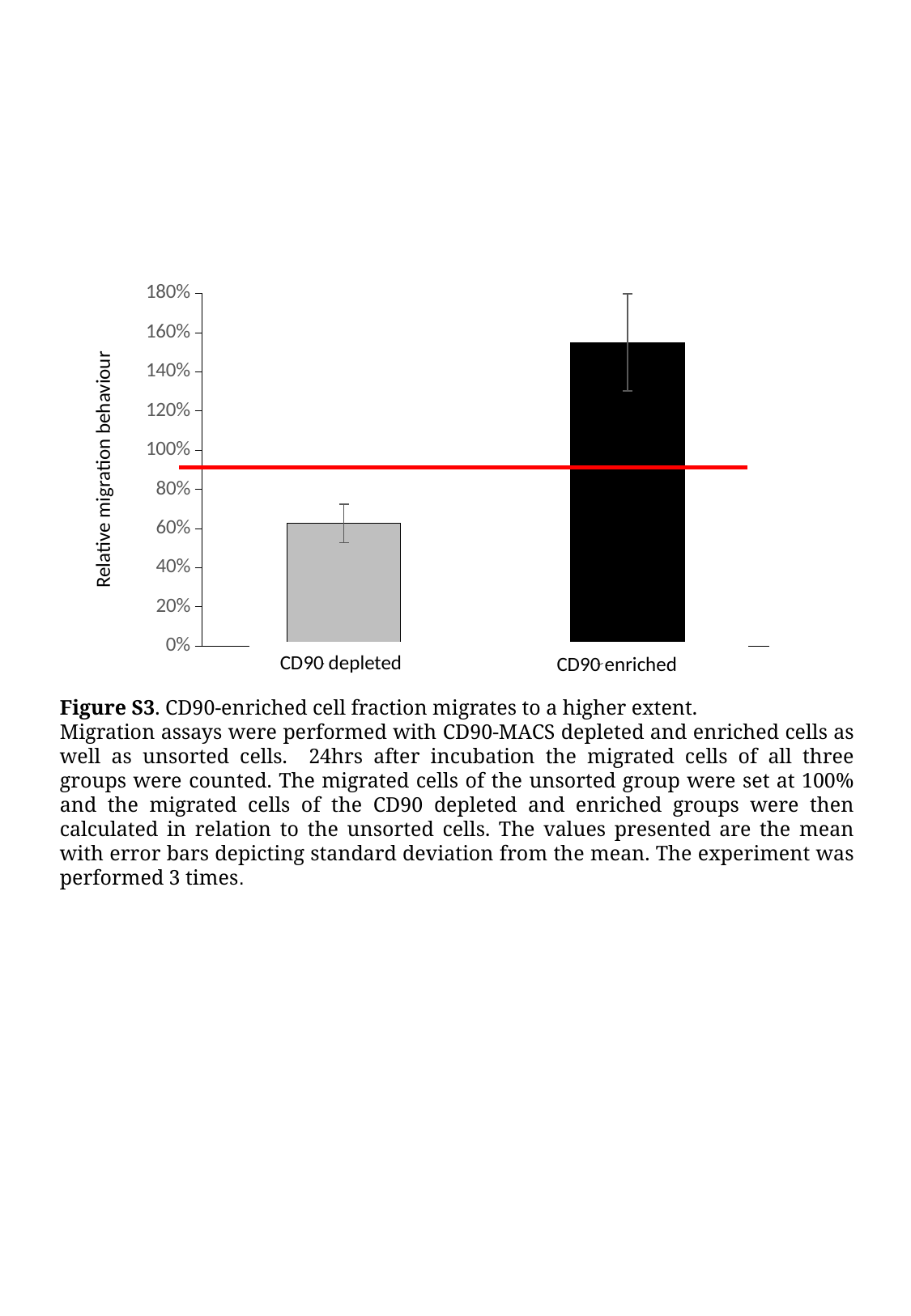

### Chart
| Category | Durchschnittliches Verhältnis zu unsortierten Zellen |
|---|---|
| CD90 depletierte Zellen | 0.6262761500509909 |
| CD90 angereicherte Zellen | 1.551060702112217 |
CD90 depleted
CD90 enriched
Relative migration behaviour
Figure S3. CD90-enriched cell fraction migrates to a higher extent.
Migration assays were performed with CD90-MACS depleted and enriched cells as well as unsorted cells. 24hrs after incubation the migrated cells of all three groups were counted. The migrated cells of the unsorted group were set at 100% and the migrated cells of the CD90 depleted and enriched groups were then calculated in relation to the unsorted cells. The values presented are the mean with error bars depicting standard deviation from the mean. The experiment was performed 3 times.

## Slide 4
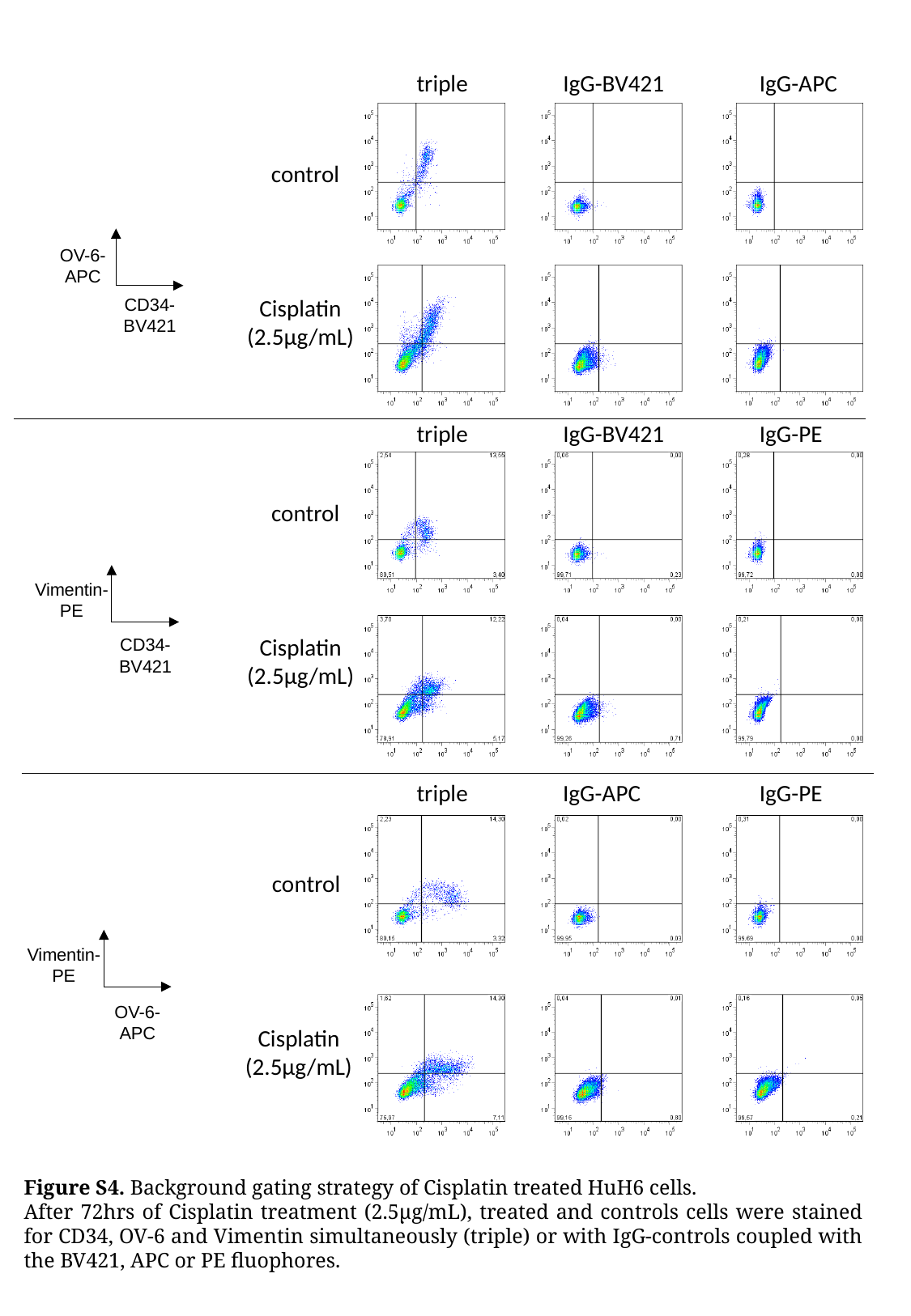

triple
IgG-BV421
IgG-APC
control
OV-6-
APC
CD34-
BV421
Cisplatin
(2.5µg/mL)
triple
IgG-BV421
IgG-PE
control
Vimentin-
PE
CD34-
BV421
Cisplatin
(2.5µg/mL)
triple
IgG-APC
IgG-PE
control
Vimentin-
PE
OV-6-
APC
Cisplatin
(2.5µg/mL)
Figure S4. Background gating strategy of Cisplatin treated HuH6 cells.
After 72hrs of Cisplatin treatment (2.5µg/mL), treated and controls cells were stained for CD34, OV-6 and Vimentin simultaneously (triple) or with IgG-controls coupled with the BV421, APC or PE fluophores.

## Slide 5
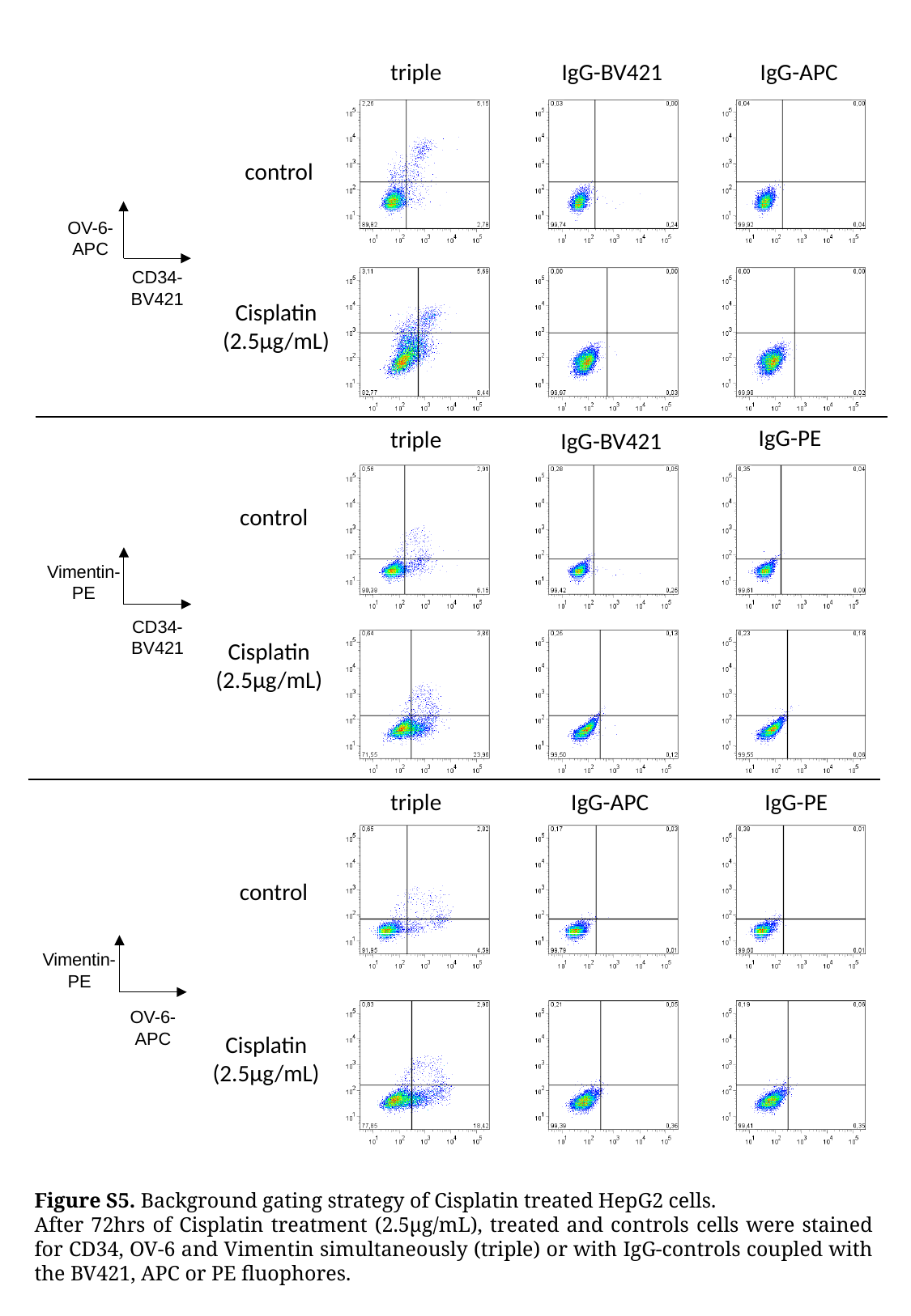

triple
IgG-BV421
IgG-APC
control
OV-6-
APC
CD34-
BV421
Cisplatin
(2.5µg/mL)
IgG-PE
triple
IgG-BV421
control
Vimentin-
PE
CD34-
BV421
Cisplatin
(2.5µg/mL)
triple
IgG-APC
IgG-PE
control
Vimentin-
PE
OV-6-
APC
Cisplatin
(2.5µg/mL)
Figure S5. Background gating strategy of Cisplatin treated HepG2 cells.
After 72hrs of Cisplatin treatment (2.5µg/mL), treated and controls cells were stained for CD34, OV-6 and Vimentin simultaneously (triple) or with IgG-controls coupled with the BV421, APC or PE fluophores.

## Slide 6
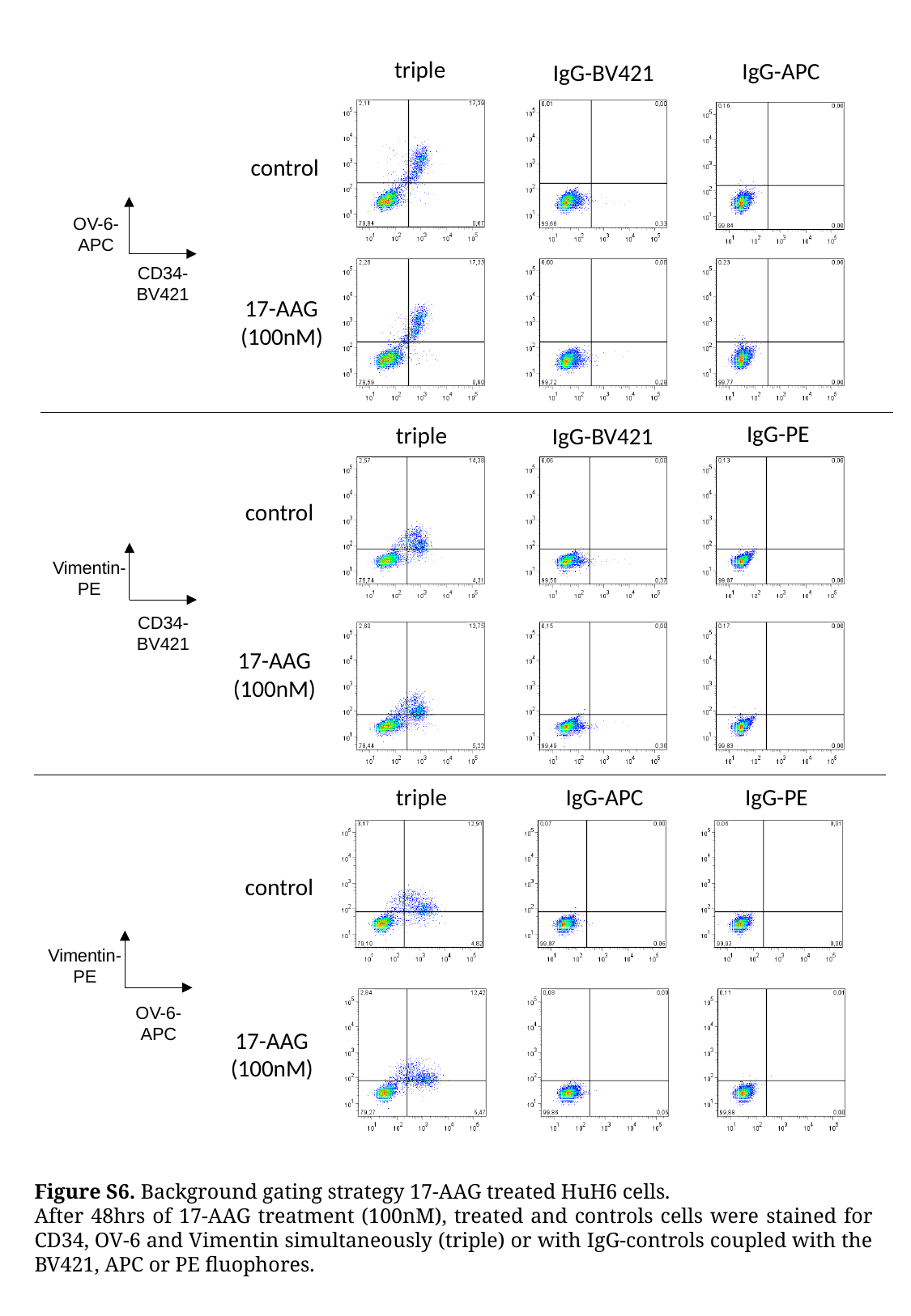

triple
IgG-APC
IgG-BV421
control
OV-6-
APC
CD34-
BV421
17-AAG
(100nM)
IgG-PE
triple
IgG-BV421
control
Vimentin-
PE
CD34-
BV421
17-AAG
(100nM)
triple
IgG-APC
IgG-PE
control
Vimentin-
PE
OV-6-
APC
17-AAG
(100nM)
Figure S6. Background gating strategy 17-AAG treated HuH6 cells.
After 48hrs of 17-AAG treatment (100nM), treated and controls cells were stained for CD34, OV-6 and Vimentin simultaneously (triple) or with IgG-controls coupled with the BV421, APC or PE fluophores.

## Slide 7
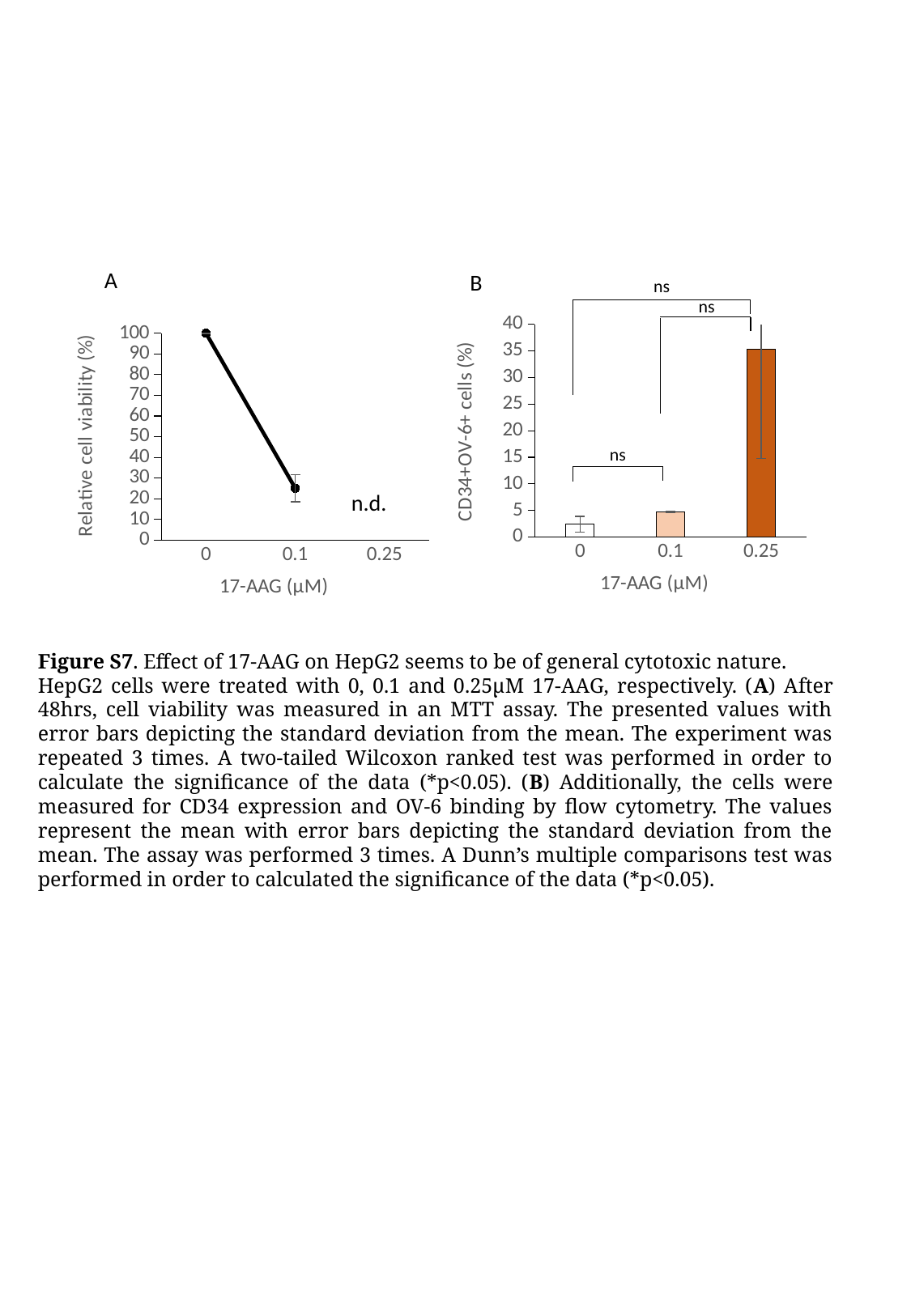

A
B
### Chart
| Category | Mittelwert |
|---|---|
| 0 | 2.3866666666666667 |
| 0.1 | 4.785 |
| 0.25 | 35.29 |
### Chart
| Category | MTT |
|---|---|
| 0 | 100.0 |
| 0.1 | 25.076550403731005 |
| 0.25 | None |n.d.
ns
ns
ns
Figure S7. Effect of 17-AAG on HepG2 seems to be of general cytotoxic nature.
HepG2 cells were treated with 0, 0.1 and 0.25µM 17-AAG, respectively. (A) After 48hrs, cell viability was measured in an MTT assay. The presented values with error bars depicting the standard deviation from the mean. The experiment was repeated 3 times. A two-tailed Wilcoxon ranked test was performed in order to calculate the significance of the data (*p<0.05). (B) Additionally, the cells were measured for CD34 expression and OV-6 binding by flow cytometry. The values represent the mean with error bars depicting the standard deviation from the mean. The assay was performed 3 times. A Dunn’s multiple comparisons test was performed in order to calculated the significance of the data (*p<0.05).

## Slide 8
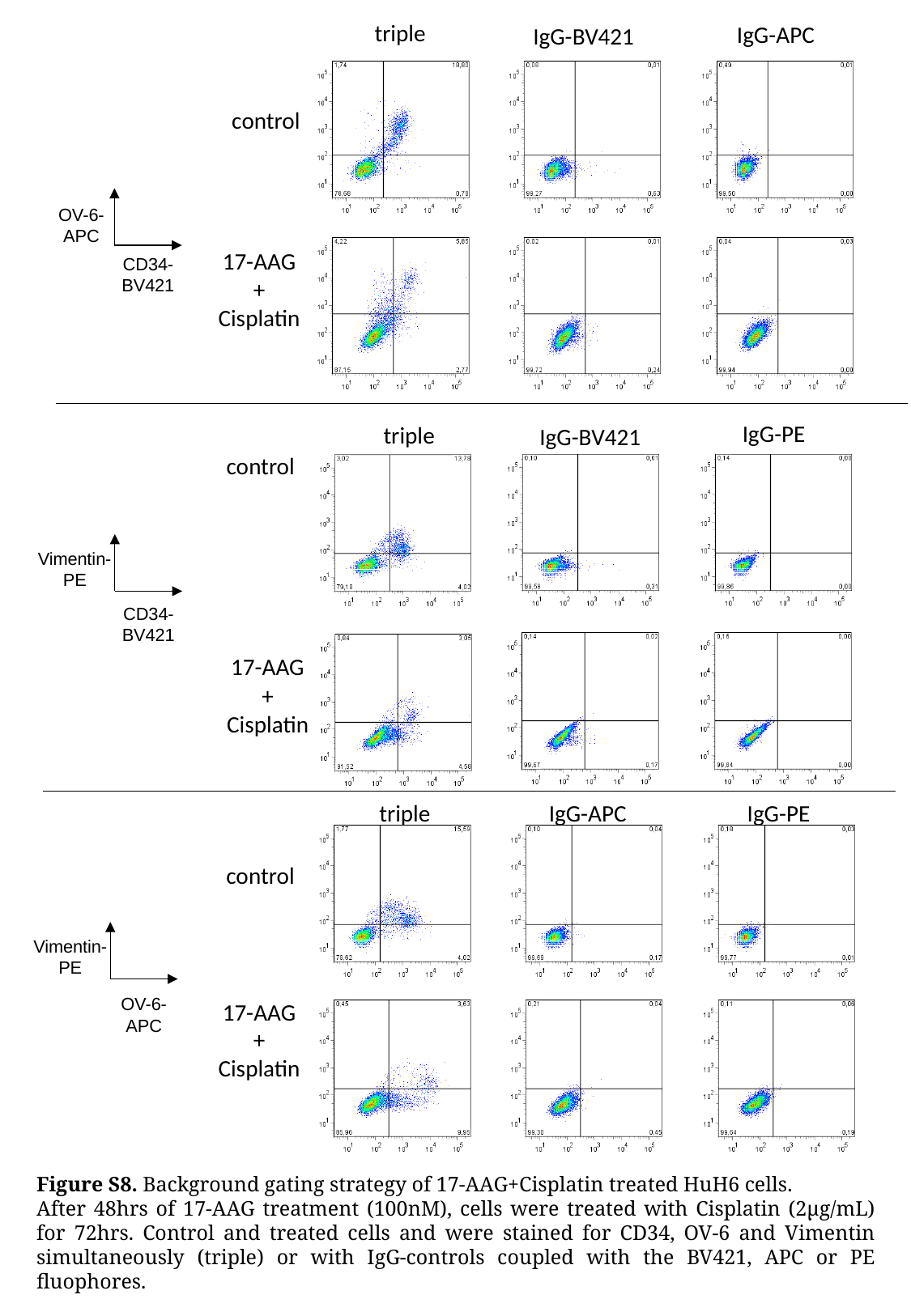

triple
IgG-APC
IgG-BV421
control
OV-6-
APC
CD34-
BV421
17-AAG
+
Cisplatin
IgG-PE
triple
IgG-BV421
control
Vimentin-
PE
CD34-
BV421
17-AAG
+
Cisplatin
triple
IgG-APC
IgG-PE
control
Vimentin-
PE
OV-6-
APC
17-AAG
+
Cisplatin
Figure S8. Background gating strategy of 17-AAG+Cisplatin treated HuH6 cells.
After 48hrs of 17-AAG treatment (100nM), cells were treated with Cisplatin (2µg/mL) for 72hrs. Control and treated cells and were stained for CD34, OV-6 and Vimentin simultaneously (triple) or with IgG-controls coupled with the BV421, APC or PE fluophores.

## Slide 9
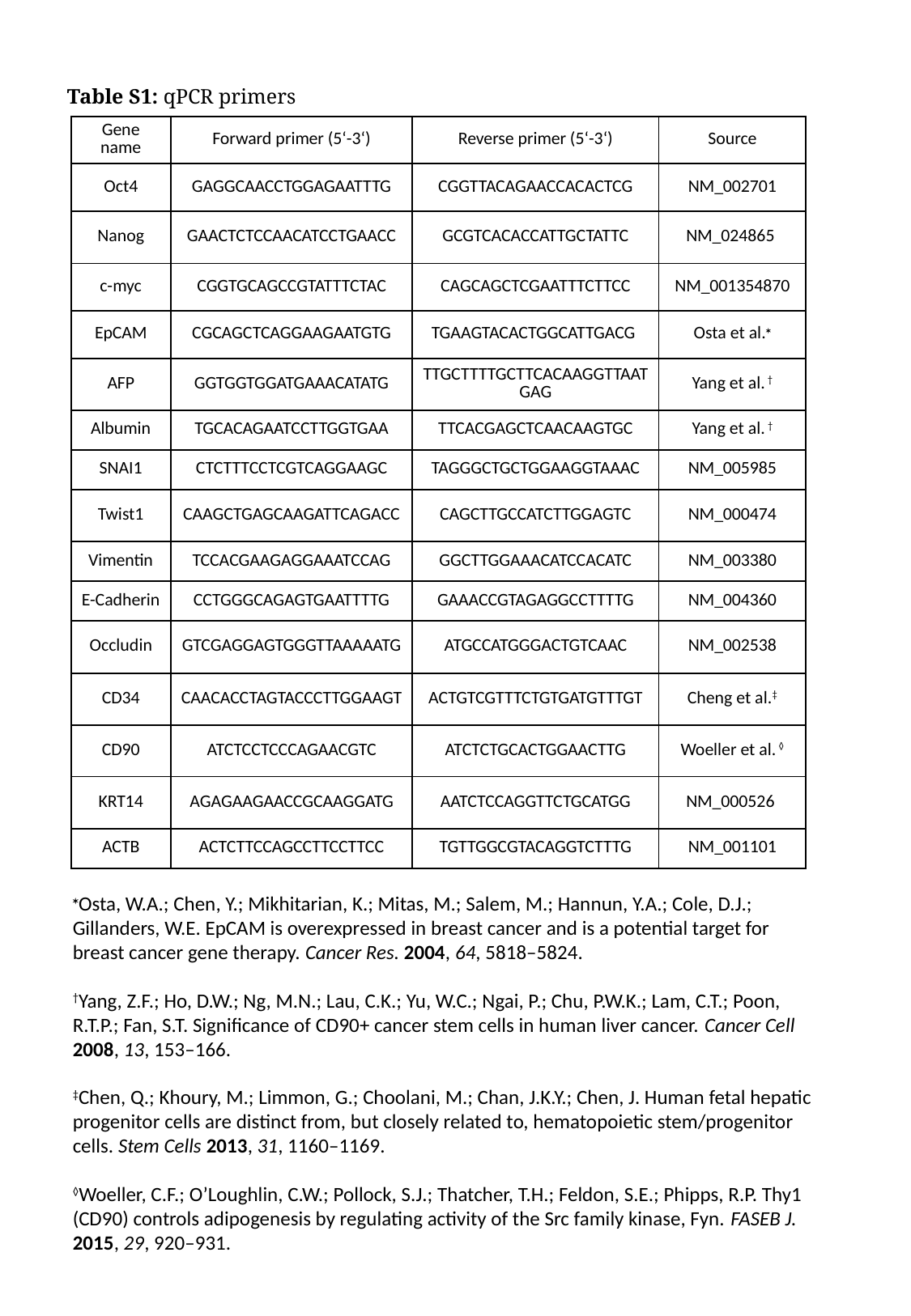

Table S1: qPCR primers
| Gene name | Forward primer (5‘-3‘) | Reverse primer (5‘-3‘) | Source |
| --- | --- | --- | --- |
| Oct4 | GAGGCAACCTGGAGAATTTG | CGGTTACAGAACCACACTCG | NM\_002701 |
| Nanog | GAACTCTCCAACATCCTGAACC | GCGTCACACCATTGCTATTC | NM\_024865 |
| c-myc | CGGTGCAGCCGTATTTCTAC | CAGCAGCTCGAATTTCTTCC | NM\_001354870 |
| EpCAM | CGCAGCTCAGGAAGAATGTG | TGAAGTACACTGGCATTGACG | Osta et al. |
| AFP | GGTGGTGGATGAAACATATG | TTGCTTTTGCTTCACAAGGTTAATGAG | Yang et al. † |
| Albumin | TGCACAGAATCCTTGGTGAA | TTCACGAGCTCAACAAGTGC | Yang et al. † |
| SNAI1 | CTCTTTCCTCGTCAGGAAGC | TAGGGCTGCTGGAAGGTAAAC | NM\_005985 |
| Twist1 | CAAGCTGAGCAAGATTCAGACC | CAGCTTGCCATCTTGGAGTC | NM\_000474 |
| Vimentin | TCCACGAAGAGGAAATCCAG | GGCTTGGAAACATCCACATC | NM\_003380 |
| E-Cadherin | CCTGGGCAGAGTGAATTTTG | GAAACCGTAGAGGCCTTTTG | NM\_004360 |
| Occludin | GTCGAGGAGTGGGTTAAAAATG | ATGCCATGGGACTGTCAAC | NM\_002538 |
| CD34 | CAACACCTAGTACCCTTGGAAGT | ACTGTCGTTTCTGTGATGTTTGT | Cheng et al.‡ |
| CD90 | ATCTCCTCCCAGAACGTC | ATCTCTGCACTGGAACTTG | Woeller et al. ◊ |
| KRT14 | AGAGAAGAACCGCAAGGATG | AATCTCCAGGTTCTGCATGG | NM\_000526 |
| ACTB | ACTCTTCCAGCCTTCCTTCC | TGTTGGCGTACAGGTCTTTG | NM\_001101 |
Osta, W.A.; Chen, Y.; Mikhitarian, K.; Mitas, M.; Salem, M.; Hannun, Y.A.; Cole, D.J.; Gillanders, W.E. EpCAM is overexpressed in breast cancer and is a potential target for breast cancer gene therapy. Cancer Res. 2004, 64, 5818–5824.
†Yang, Z.F.; Ho, D.W.; Ng, M.N.; Lau, C.K.; Yu, W.C.; Ngai, P.; Chu, P.W.K.; Lam, C.T.; Poon, R.T.P.; Fan, S.T. Significance of CD90+ cancer stem cells in human liver cancer. Cancer Cell 2008, 13, 153–166.
‡Chen, Q.; Khoury, M.; Limmon, G.; Choolani, M.; Chan, J.K.Y.; Chen, J. Human fetal hepatic progenitor cells are distinct from, but closely related to, hematopoietic stem/progenitor cells. Stem Cells 2013, 31, 1160–1169.
◊Woeller, C.F.; O’Loughlin, C.W.; Pollock, S.J.; Thatcher, T.H.; Feldon, S.E.; Phipps, R.P. Thy1 (CD90) controls adipogenesis by regulating activity of the Src family kinase, Fyn. FASEB J. 2015, 29, 920–931.
